# The Importance of Intraoperative Selenium Blood Levels on Organ Dysfunction in Patients Undergoing Off-Pump Cardiac Surgery: A Randomised Controlled Trial

**DOI:** 10.1371/journal.pone.0104222

**Published:** 2014-08-13

**Authors:** Ana Stevanovic, Mark Coburn, Ares Menon, Rolf Rossaint, Daren Heyland, Gereon Schälte, Thilo Werker, Willibald Wonisch, Michael Kiehntopf, Andreas Goetzenich, Steffen Rex, Christian Stoppe

**Affiliations:** 1 Department of Anaesthesiology, University Hospital of the RWTH Aachen, Aachen, Germany; 2 Department of Thoracic, Cardiac and Vascular Surgery, University Hospital, RWTH Aachen, Aachen, Germany; 3 Kingston General Hospital, Kingston, Ontario, Canada; 4 Institute of Physiological Chemistry, Centre for Physiological Medicine, Medical University of Graz, Graz, Austria; 5 Clinical Institute of Medical and Chemical Laboratory Diagnostics, Medical University of Graz, Graz, Austria; 6 Institute of Clinical Chemistry, Friedrich-Schiller University, Jena, Germany; 7 Department of Anaesthesiology and Department of Cardiovascular Sciences, University Hospitals Leuven, KU Leuven, Belgium; 8 Institute of Biochemistry and Molecular Cell Biology, RWTH Aachen University, Aachen, Germany; San Raffaele Scientific Institute, Italy

## Abstract

**Introduction:**

Cardiac surgery is accompanied by an increase of oxidative stress, a significantly reduced antioxidant (AOX) capacity, postoperative inflammation, all of which may promote the development of organ dysfunction and an increase in mortality. Selenium is an essential co-factor of various antioxidant enzymes. We hypothesized a less pronounced decrease of circulating selenium levels in patients undergoing off-pump coronary artery bypass (OPCAB) surgery due to less intraoperative oxidative stress.

**Methods:**

In this prospective randomised, interventional trial, 40 patients scheduled for elective coronary artery bypass grafting were randomly assigned to undergo either on-pump or OPCAB-surgery, if both techniques were feasible for the single patient. Clinical data, myocardial damage assessed by myocard specific creatine kinase isoenzyme (CK-MB), circulating whole blood levels of selenium, oxidative stress assessed by asymmetric dimethylarginine (ADMA) levels, antioxidant capacity determined by glutathionperoxidase (GPx) levels and perioperative inflammation represented by interleukin-6 (IL-6) levels were measured at predefined perioperative time points.

**Results:**

At end of surgery, both groups showed a comparable decrease of circulating selenium concentrations. Likewise, levels of oxidative stress and IL-6 were comparable in both groups. Selenium levels correlated with antioxidant capacity (GPx: r = 0.720; p<0.001) and showed a negative correlation to myocardial damage (CK-MB: r = −0.571, p<0.001). Low postoperative selenium levels had a high predictive value for the occurrence of any postoperative complication.

**Conclusions:**

OPCAB surgery is not associated with less oxidative stress and a better preservation of the circulating selenium pool than on-pump surgery. Low postoperative selenium levels are predictive for the development of complications.

**Trial registration:**

ClinicalTrials.gov NCT01409057

## Introduction

In cardiac surgery, the use of cardioplegic cardiac arrest and cardiopulmonary bypass (CPB) is known to trigger a significant release of reactive oxygen and nitrogen species (ROS, RNOS) [1]. Termination of cardioplegic arrest by reperfusion leads to oxidative stress, which is a major contributor to the complex pathophysiology of ischaemia-reperfusion injury (I/R) [2], [3]. Moreover, extracorporeal circulation (ECC) [4] by itself is known to stimulate the production of ROS in neutrophils and monocytes [5]. When exceeding the endogenous antioxidant (AOX) capacity [6], oxidative stress results in the oxidation of proteins, membrane lipids and deoxyribonucleic acids [3], [7]. Although often remaining sub-clinical and resolving promptly, postoperative inflammation and oxidative stress can contribute to the development of the systemic inflammatory response syndrome, which is frequently observed after cardiac surgery and may further progress to multiple organ dysfunctions (MOD) and eventual death of patients [2], [8]. Amongst the various endogenous AOX defence lines, selenium plays a unique role as essential co-factor for different AOX-enzymes that are involved in the detoxification of both ROS and RNOS [9]. We recently demonstrated that patients undergoing cardiac surgery with cardiopulmonary bypass (CPB) and cardioplegic arrest showed a significant intraoperative decrease in circulating selenium levels, which was independently associated with the postoperative development of MOD [10]. The underlying mechanisms for the decrease in selenium levels have not been comprehensively elaborated yet. It is known from other antioxidants that their circulating concentrations are depleted when scavenging reactive oxygen species during/after CPB [11]. Furthermore, selenium might be trans located into the interstitial compartment during inflammation [12], [13] and/or might be adsorbed by an extracorporeal circuit [14]. Although off-pump coronary artery bypass grafting (OPCAB) has become increasingly popular in selected patients, the effects of OPCAB-surgery on perioperative circulating selenium levels have not been thoroughly studied yet. Due to the abstinence of cardioplegic arrest and preservation of normothermia, OPCAB-surgery should theoretically be associated with less oxidative stress [6], [15]. In contrast, various studies repeatedly demonstrated for OPCAB-surgery a systemic inflammation that is comparable to on-pump cardiac surgery [16], [17]. Comparing on-pump with OPCAB patients should therefore allow to distinguish the effects of oxidative stress from those of inflammation on circulating selenium levels.

Therefore we analysed perioperative selenium levels, the overall inflammatory response and oxidative stress in patients undergoing OPCAB-surgery in comparison to patients that underwent on-pump coronary artery bypass cardiac surgery (CABG). We hypothesized a less pronounced decrease of circulating selenium levels in the off-pump group, owing to less intraoperative oxidative stress than in the on-pump group. This is an additional analysis of data collected in cardiac surgical patients in which the effects of on- and off-pump surgery on the release of macrophage migration inhibitory factor have been previously been reported [18].

## Methods

### Study design and patients

This mono-centre study was designed as a randomised, interventional clinical trial at the University Hospital of the RWTH Aachen, Germany. It was registered at ClinicalTrials.gov (NCT01409057). The protocol for this trial and supporting CONSORT checklist are available as supporting information; see Checklist S1 and Protocol S1. Data on the perioperative release of Macrophage Migration Inhibitory Factor (MIF) obtained from the same patients have been recently published elsewhere [18].

**Figure 1:**
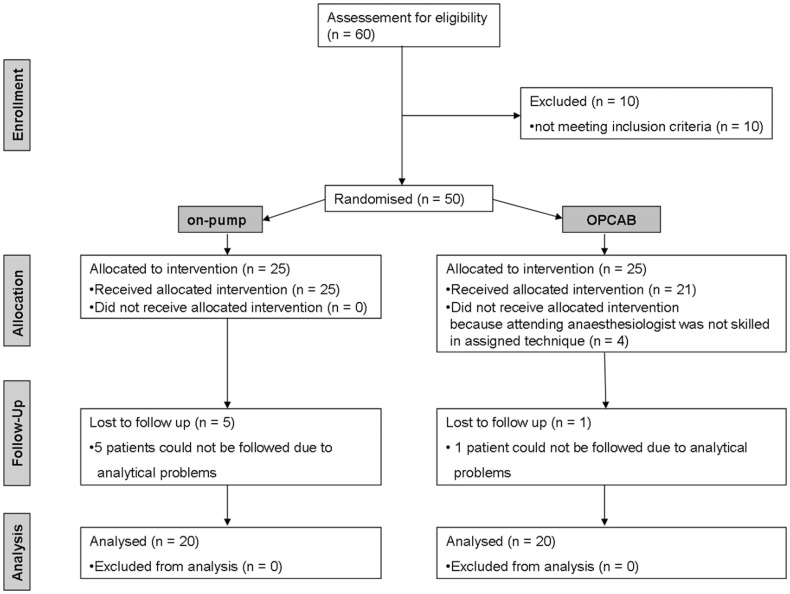
Flowchart. According to the *CONSORT-statement* for randomised clinical trials. From the initially screened 60 patients, 46 patients received the allocated intervention. 6 patients had to be excluded from further analysis.

After approval of the institutional review board (*Ethics commission RWTH Aachen, EK 086/10*), written informed consent was obtained. We initially screened for enrolling a total of 60 patients scheduled for elective isolated CABG. From 50 randomised patients, a total of 40 were followed until final analysis. We included only patients ≥18 years. Exclusion criteria were severe hepatic and renal failure (serum creatinine >200 µmol l^−1^) and patients for whom either on- or off-pump techniques were not considered feasible. Furthermore patients with a severe ischemic cardiomyopathia, a recent (<7 days) myocardial infarction and an emergency operation were excluded. Preoperatively, the attending surgeon assessed the eligibility of the potential study participants for off-pump coronary artery bypass grafting. After obtainment of written informed consent, eligible patients were then randomised by a closed envelope technique into either the on-pump or into the OPCAB-group (Fig. 1). Of note, the randomisation list has been created prior to the start of study. The investigators who assessed postoperative outcome remained blinded throughout the whole study.

### Anaesthesia

As usual in our institution, anaesthesia was induced by etomidate (0.1–0.2 mg·kg^−1^) and sufentanil (0.5–1 µg·kg^−1^). The patient was endotracheally intubated after application of rocuronium (1 mg·kg^−1^). General anaesthesia was maintained with sevoflurane (0.6–1.0%) and sufentanil (0.5–1 µg·kg^−1^h^−1^). Balanced crystalloid solutions 1 ml·kg^−1^·h^−1^ were used to manage the fluid balance. Upon the end of the surgery all patients were admitted to the ICU.

### Surgical procedure

All patients underwent a midline sternotomy. For performing the bypasses, the internal mammaria artery was harvested as a pedicle. Moreover, venous conduits were used. Heparin was administered to achieve an activated clotting time (ACT) of >400 s (on-pump) and 250–300 s (OPCAB) and antagonized in proportion of 1:1 with protamine at end of surgery. The patients' temperatures were either maintained normothermic (OPCAB) or had a minimum of 32°C (on-pump) during CPB.

#### On-pump CABG

CPB was performed on a conventional CPB circuit. Cardiac arrest was generated by the antegrade infusion of cold crystalloid cardioplegic solution. A nonpulsatile pump flow of 2.2 L min^−1^·m^−2^ was maintained throughout CPB.

#### OPCAB

Patients were operated in a right rotated Trendelenburg position. To prevent myocardial ischaemia during placement of the distal anastomoses, an intracoronary shunt was inserted. To facilitate the performance of distal anastomosis, commercially available mechanical stabilizers were used.

### Hemodynamic management

The intraoperative hemodynamic management was according to our clinical routine. Hypovolaemia was treated with colloid solutions (hydroxyethylstarch 130/0.4, Voluven, Fresenius Kabi, Bad Homburg, Germany). The threshold value for transfusion of packed red blood cells (PRBC) was a haemoglobin content <7.5 g·dl^−1^. If further haemodynamic stabilization was necessary, norepinephrine was administered. If required, epinephrine was applied for inotropic support.

### Data collection

Preoperatively, we documented relevant medical data and baseline characteristics. According to the ACCP/SCCM consensus conference criteria [19], we recorded during the observation period the duration of mechanical ventilation, the ICU- and hospital length of stay and the incidence of systemic inflammatory response syndrome (SIRS), sepsis, severe sepsis and septic shock. Furthermore, the incidence of any organ dysfunction was evaluated by established organ failure variables [19]. In addition, organ dysfunction was assessed on the 1^st^ postoperative day (1.POD) by the means of the simplified acute physiology score (SAPS II) [20] and the sequential organ failure assessment (SOFA) [21].

### Laboratory assessment

Serum and whole blood probes for the measurement of selenium, GPX and markers of oxidative stress were drawn from the central venous catheter after induction of anaesthesia and at ICU-admission. Whole blood samples were stored at room temperature and serum samples were immediately stored at −80°C until final analysis.

Electrothermal atomic absorption spectroscopy (ASS) (5100 PC, Perkin-Elmer, Paris France) was used to determine whole blood selenium-concentrations [22].

We measured serum levels of interleukin-6 (IL-6), to assess the inflammatory response [23] by a commercially available enzyme-linked immunosorbent assay (ELISA) kit (IL-6, R&D Systems, Minneapolis, MN, USA).

The myocard specific creatine kinase isoenzyme (CK-MB) was analyzed by a centrifugal analyzer (Cobas 8000, Roche, Switzerland) to evaluate the extent of myocardial damage [24], [25].

The GPx-activity was assessed in serum. Reduction of oxidized glutathione (GSH) was coupled with a peroxidase reaction and one unit of GPx-activity leads to oxidation of 1 mol NADPH min^−1^ [26]. Selenium and GPx-activities were determined in the laboratories of biosyn Arzneimittel GmbH, Fellbach, Germany and GPx-activities in addition in the Institute of Clinical Chemistry, Friedrich-Schiller Universität Jena, Germany.

Serum levels of ADMA were measured using an ADMA ELISA Kit [27].

### Statistical analysis

Statistical analysis was performed with a commercially available software package (SPSS 21.0 (IBM Corporation, Armonk, NY, USA).

Originally, we had planned to include 100 patients in this study as a pre-study power-analysis was not possible due to a complete lack of available data in the literature. Unfortunately, the cooperating surgeon unexpectedly left our institution before the enrolment of patients was terminated. At that time 46 patients had been enrolled. All participating investigators discussed whether it is possible to continue the study under these circumstances. Since we could no longer assure comparable conditions for all patients we decided to stop the recruitment of patients and close the study. Due to analytical problems and missing outcome data, only 40 patients of the 46 randomised patients were analysed per modified ITT-analysis according to the CONSORT-recommendations. A power analysis on the basis of the so far obtained data, using nQuery Advisor Version 7.0 (Statistical Solutions, Saugus, Massachusetts, USA), was performed to get an impression of how large the probability for a type II error is. This analysis revealed that the observed difference from pre- to postoperative selenium concentrations in the OPCAB and on-pump group had a statistical power of greater than 80% with a significance level of 0.05. The decision to close the study was not influenced by the results of the power analysis.

Our primary endpoint was the difference in selenium decrease during two different techniques of coronary artery bypass grafting.

As secondary endpoints, we investigated the association between circulating selenium levels and the extent of oxidative stress as reflected by ADMA and GPx. The degree of perioperative inflammation was assessed by serum levels of IL-6. Furthermore, we evaluated clinically relevant outcome parameters involving SAPS II and SOFA score, duration of mechanical ventilation, the hospital- and ICU-length of stay. The degree of perioperative myocardial damage was assessed by CK-MB. The occurrence of postoperative complications was assessed separately with respect to the single organs and as composite outcome, which evaluated the occurrence of organ dysfunction and death.

Normal distribution was tested by the Shapiro-Wilk *W*-test. We compared single measurements, if with the Students *t*-test. A two-way ANOVA was used to compare the results of repeated measurements to take into account the correlated observations within the groups. We included as fixed effects the grouping factor treatment (OPCAB vs. on-pump) and the within-factor time. We used the Mann–Whitney *U* test for nonparametric data. Significant results were post hoc tested with the Bonferroni adjustment for multiple measurements (not normally distributed data), respectively. The Fisher's exact test was used to compare proportions of data with an incidence of <5, the Chi-square test was used for incidences >5.

The predictive value of selenium concentrations for the occurrence of organ dysfunction was determined by calculating the area under the curve (AUC) of the receiver-operating characteristic curves (ROC). *P-*values <0.05 were considered statistically significant in all statistical analyses.

## Results

### Enrolled patients

From sixty screened patients scheduled for CABG-surgery, fifty patients fulfilled all inclusion criteria and were randomised between June 2010 and December 2012 (Fig.1). We performed a modified ITT-analysis of 40 patients. The enrolled patients were also part of the previously published trial of our group, which analysed the significance of perioperative release of macrophage inhibitory factor (MIF) [18]. Preoperative baseline patient characteristics did not show any significant differences between the two groups (table 1).

**Table 1:** Patient baseline characteristics and data on surgery in the two groups.

		All patients (n = 40)			Groups							
					on-pump (n = 20)				OPCAB (n = 20)			*p-value*
**Demographic Data**				**[95% CI]**				**[95% CI]**			**[95% CI]**	
Age	years	67±10		[64–70]	67±12			[61–72]	67*±*9		[63–71]	*0.787*
Sex, male	n (%)	32 (80)			16(80)				16 (80)			*1.000*
Height	cm	172±10		[169–175]	171±10			[166–175]	173±9		]168–177]	*0.524*
Weight	kg	82±14		[77–86]	82±13			[75–88]	82±15		[75–89]	*0.896*
euroSCORE		5±3		[5]–[6]	5±2			[4]–[7]	5±3		[4]–[7]	*0.857*
**Prior or pre-existing disease**												
Hypertension	n (%)	31 (78)			17 (85)				14 (70)			*0.451*
Chronic pulmonary disease	n (%)	10 (25)			7 (35)				3 (15)			*0.144*
Extra cardiac arteriopathy	n (%)	14 (35)			7 (35)				7 (35)			*1.000*
Cerebral dysfunction	n (%)	3 (7.5)			2 (10)				1 (5)			*1.000*
Unstable angina	n (%)	11 (28)			5 (13)				6 (15)			*0.723*
Recent myocardial infarction (<90d)	n (%)	14 (35)			7 (35)				7 (35)			*1.000*
Chronic kidney disease	n (%)	7 (18)			3 (15)				4 (20)			*1.000*
Liver disease	n (%)	1 (2.5)			0 (0)				1 (5)			*1.000*
Diabetes	n (%)	6 (15)			3 (15)				3 (15)			*1.000*
LVEF > 50%	n (%)	31 (78)			17 (85)				14 (70)			*0.451*
LVEF 30 - 50%	n (%)	7 (18)			3 (15)				4 (20)			*1.000*
LVEF < 30%	n (%)	2 (5)			0 (0)				2 (10)			*0.487*
**Intraoperative data**												
Intraoperative fluid balance	ml	2413±1146		[2007–2819]	2890±1077			[2337–3444]	1906±1016*		[1365–2447]	*0.011*
PRBC	n	0 (0–5)			0 (0–5)				0 (0–4)			*0.416*
Fluid balance within first 24h	Ml	2673±1313		[2248–3099]	2810±1340			[2183–3437]	2529±1305		[1900–3158]	*0.511*
Haemoglobin at admission	g/dl	10±1		[10–10]	10±1			[9]–[10]	10±1		[9]–[10]	*0.094*
Duration of surgery	min	214±54		[196–231]	230±51			[206–254]	198±54		[172–223]	*0.058*
Ischaemia Time	min				56±20			[46–66]			n.a.	
Time of recirculation	min				32±11			[26]–[37]			n.a.	
CPB Time	min				100±28			[87–113]			n.a.	

Data are presented as median (range) (not normally distributed data), as mean ± SD (normally distributed data) or as

absolute numbers (with the percentage (%) of the whole). * p<0.05

[95% CI]  =  95% Confidence intervall on the mean

CABG  =  coronary artery bypass grafting; CPB  =  cardiopulmonary bypass, MI  =  myocardial infarction, PRBC = packed red blood cells.

### Perioperative selenium-levels

**Figure 2:**
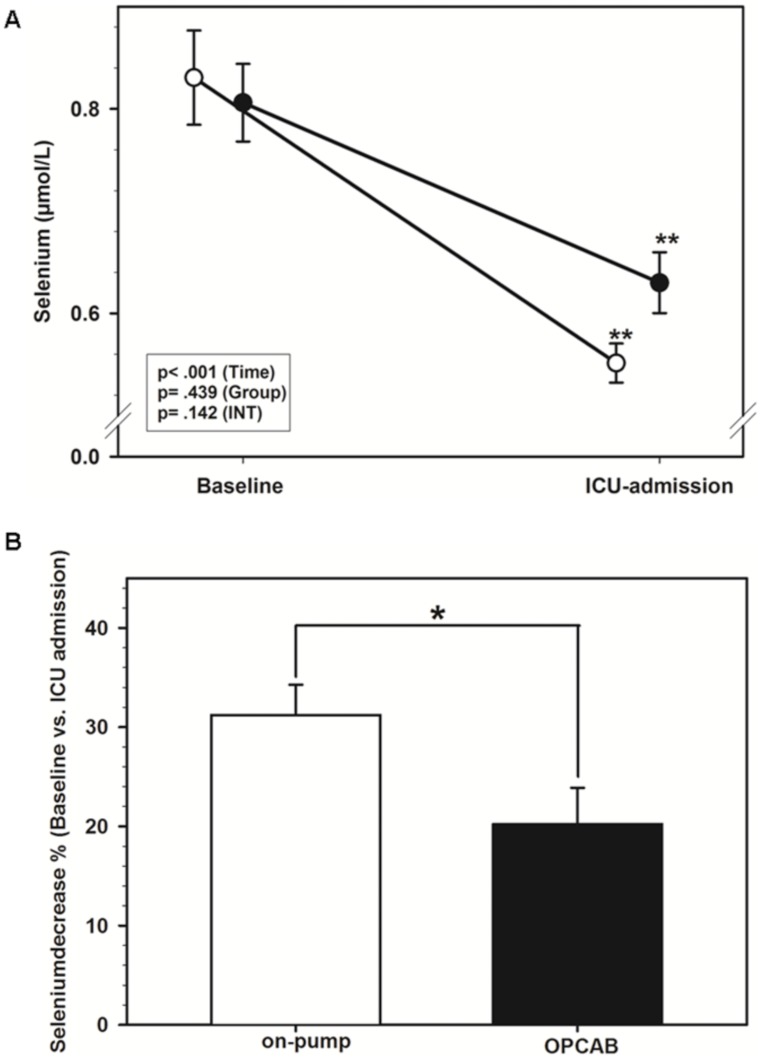
Perioperative selenium-levels. **A**) Comparison of whole blood selenium levels between the on-pump group (open circles) and the OPCAB-group (closed circles) at baseline (preoperative) and at ICU admission (postoperative). Data are presented as mean ± standard deviation. *p<0.05, **p<0.01 versus baseline, analyzed with 2-way ANOVA. **B**) Comparison of the intraoperative percentual decrease of whole blood selenium between the on-pump group (white bar) and the OPCAB-group (black bar). *p<0.05, **p<0.01 between the two groups, analyzed with the Mann–Whitney U test.

Baseline selenium values were comparable in both study groups and lower than the European reference value of 100–140 µg l^−1^ [28], [29]. Both groups demonstrated a significant and comparable intraoperative decrease of circulating selenium levels (Fig. 2A). The extent of decrease, measured with the Mann–Whitney *U* test, was more pronounced in the on-pump group (31.2±13.6 (mean ± SD) % vs. 20.2±16.3%; p = 0.040) (Fig. 2B).

### Perioperative time course of markers of oxidative stress and antioxidant capacity

**Figure 3:**
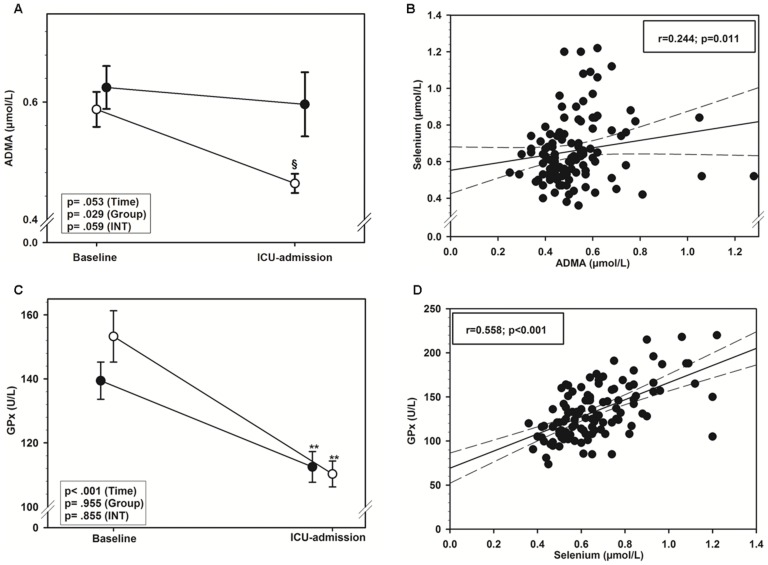
Perioperative time course of markers of oxidative stress and antioxidant capacity. **A**) Comparison of the intraoperative decrease of ADMA levels in serum between the on-pump group (open circles) and the OPCAB-group (closed circles) at baseline (preoperative) and at ICU admission (postoperative). Data are presented as mean ± standard deviation. ^§^ *p*<0.05, ^§§^ *p*<0.01 versus OPCAB group, analyzed with 2-way ANOVA. **B**) Correlation of whole blood selenium levels and ADMA in serum between the two groups. Data are depicted as linear regression (black line) with 95% confidence intervals (long dashed line). **C**) Comparison of GPx levels between the on-pump group (open circles) and the OPCAB-group (closed circles) at baseline (preoperative) and at ICU admission (postoperative). Data are presented as mean ± standard deviation. *p<0.05, **p<0.01 versus baseline, analyzed with 2-way ANOVA. **D**) Correlation of whole blood selenium and GPx content in serum between the two groups. Data are depicted as linear regression (black line) with 95% confidence intervals (long dashed line).

Time course of ADMA-levels was comparable in both groups. Intraoperatively, there was a decrease (although statistically not significant) only in the on-pump group (Fig. 3A). Within the OPCAB group, ADMA levels remained unchanged throughout surgery (Fig. 3A). Circulating selenium levels were directly correlated with ADMA levels (Fig. 3B).

GPx-activity was significantly reduced in both groups after termination of surgery (Fig. 3C). The extent of intraoperative decrease showed a trend towards a significant higher reduction in the on-pump group when compared to the OPCAB (26.1±11.6% (mean *±* SD*)* vs. 19.1±15.6%; p = 0.121). Circulating selenium levels and GPx were significantly correlated (Fig. 3D).

### Perioperative inflammatory response and myocardial damage

**Figure 4:**
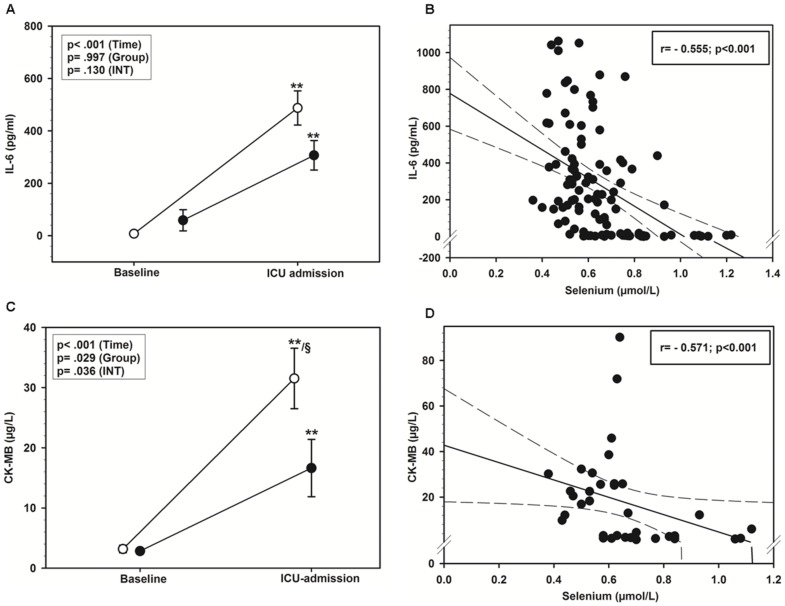
Perioperative inflammatory response and myocardial damage. **A**) Comparison of serum IL-6 levels between the on-pump group (open circles) and the OPCAB-group (closed circles) at baseline (preoperative) and at ICU admission (postoperative). Data are presented as mean ± standard deviation. *p<0.05, **p<0.01 versus baseline, analyzed with 2-way ANOVA. **B**) Correlation of whole blood selenium levels and IL-6 levels in serum, between the two groups. Data are depicted as linear regression (black line) with 95% confidence intervals (long dashed line). **C**) Comparison of serum CK-MB levels between the on-pump group (open circles) and the OPCAB-group (closed circles) at baseline (preoperative) and at ICU admission (postoperative). Data are presented as mean ± standard deviation. *p<0.05, **p<0.01 versus baseline, analyzed with 2-way ANOVA. **D**) Correlation of whole blood selenium levels and CK-MB in serum between the two groups. Data are depicted as linear regression (black line) with 95% confidence intervals (long dashed line).

The time course of perioperative IL-6 levels showed a comparable increase in both groups (Fig. 4A). Circulating selenium levels demonstrated a negative correlation to the IL-6 values within the entire observation period (Fig. 4B).

CK-MB showed a significant intraoperative increase in both groups (Fig. 4C) with a significantly higher percentual increase in the on-pump group (1195.9±1230.3% (mean ± SD) vs. 478.8±582.1; p = 0.044). We found an inverse correlation between circulating selenium- and CK-MB-levels (Fig. 4D).

### Biomarkers and postoperative organ dysfunction

**Figure 5:**
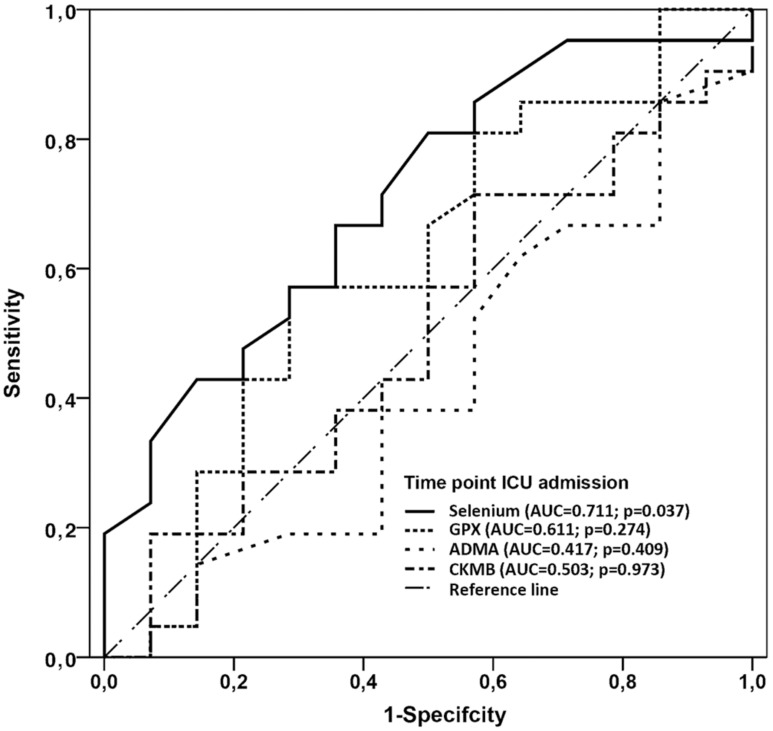
Receiver operating characteristic curve (all patients). Receiver operating characteristic curve for the significance of postoperative (admission to ICU) selenium, GPx, ADMA and CK-MB concentrations in all patients to predict the development of organ dysfunction in the postoperative period. AUC, area under the receiver operating curve.

**Figure 6:**
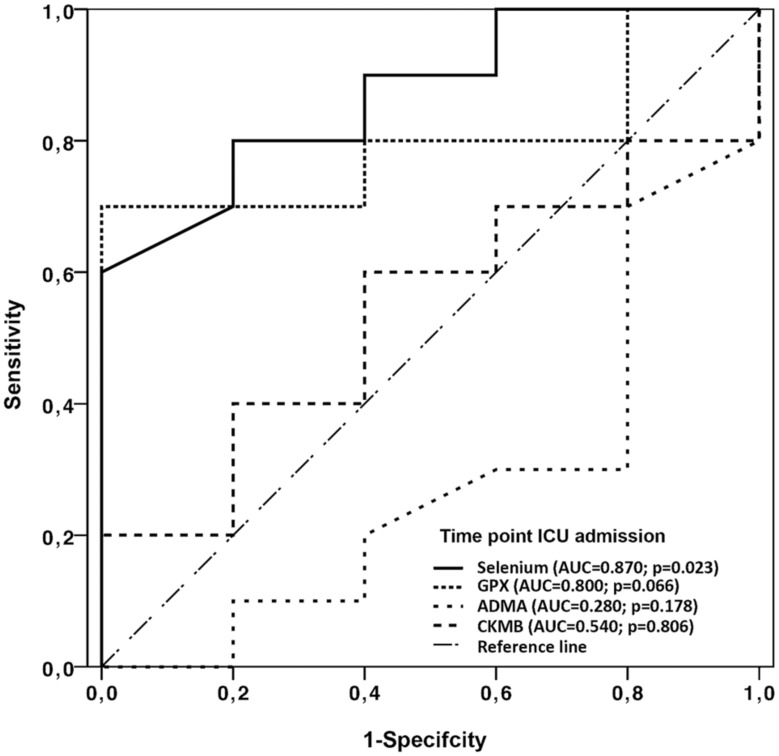
Receiver operating characteristic curve (OPCAB group). Receiver operating characteristic curve for the significance of postoperative (admission to ICU) selenium, GPx, ADMA and CK-MB concentrations in the OPCAB group to predict the development of organ dysfunction in the postoperative period. AUC, area under the receiver operating curve.

Postoperative complications and organ dysfunction are shown in table 2. No significant differences were detected between the groups. In comparison with GPX, ADMA and CKMB, only postoperative measured selenium levels had predictive accuracy for the development of postoperative organ dysfunction and death in the later time course (Fig. 5, 6).

**Table 2:** Outcome characteristics of the two groups.

		All patients (n = 40)				Groups								
						on-pump (n = 20)				OPCAB (n = 20)				*p-value*
**Postoperative organ failure/complication**					**[95% CI]**				**[95% CI]**				**[95% CI]**	
Atrial fibrillation	n (%)	6 (15)				1 (5)				5 (25)				*0.182*
Stroke	n (%)	1 (3)				0 (0)				1 (5)				*1.000*
Delir	n (%)	6 (15)				2 (10)				4 (20)				*0.661*
Acute Kidney Injury	n (%)	3 (8)				0 (0)				3 (15)				*0.231*
Pneumonia	n (%)	6 (15)				4 (20)				2 (10)				*0.661*
Cardiogenic shock	n (%)	1 (3)				0 (0)				1 (5)				*1.000*
Wound infections	n (%)	1 (3)				1 (5)				0 (0)				*1.000*
SOFA score 1.POD	n	6 (3–11)				6 (3–9)				6 (3–11)				*0.719*
SAPS II 1.POD	n	29 (18–39)				29 (18–38)				29 (21–39)				*0.951*
**Incidence of SIRS/Sepsis**														
SIRS	n (%)	14 (35)				6 (30)				8 (40)				*0.507*
Severe SIRS and Sepsis	n (%)	1 (3)				1 (5)				0 (0)				*1.000*
Septic shock	n (%)	3 (8)				1 (5)				2 (10)				*1.000*
**Longterm - Outcome**														
Sedation	hours	10±8			[7]–[12]	9±5			[7]–[11]	11±10			[6]–[15]	*0.539*
Duration of mechanical ventilation	hours	12±9			[9]–[15]	11±5			[9]–[14]	13±12			[7]–[19]	*0.586*
ICU stay	hours	80±108			[45–115]	67±90			[25–109]	93±126			[33–154]	*0.459*
Hospital length of stay	days	15±10			[12]–[18]	14±11			[9]–[20]	16±8			[12]–[20]	*0.635*
Mortality	n (%)	1 (3)				0 (0)				1 (5)				*1.000*

Data are presented as median (range) (not normally distributed data), as mean ± SD (normally distributed data) or as absolute numbers (with the percentage (%) of the whole). CABG  =  coronary artery bypass grafting; CPB  =  cardiopulmonary bypass, MI  =  myocardial infarction.

## Discussion

In the present trial, we observed an intraoperative decrease of circulating selenium levels independent from the type of cardiac surgery. The extent of oxidative stress and inflammatory response was comparable in both groups. Of note, postoperative circulating selenium levels showed a significant predictive accuracy for the occurrence of postoperative organ dysfunction.

In accordance with our previous observations [10], [30], the majority of the enrolled patients exhibited significantly reduced selenium levels already *prior* to surgery [28]. These preoperative low selenium-levels were significantly decreased in both groups during surgery, but the extent of this decrease was significantly higher in the on-pump group. We showed recently that the extent of perioperative selenium decrease after on-pump surgery was independently associated with postoperative occurrence of organ dysfunction, indicating a pivotal role of selenium within the antioxidant and anti-inflammatory defence mechanisms [10], [30]. In the present trial, we could translate these findings to patients that underwent OBCAB surgery. As low postoperative selenium levels were predictive for the occurrence of any organ dysfunction, this might indicate a key role of circulating selenium in cardiac surgical patients *per se*. Our data might also demonstrate the potential usefulness of routinely measuring the preoperative and postoperative selenium status. Due to the small sample size of our trial we cannot prove that high selenium levels may protect from adverse events. This can only be tested in a large randomised controlled trial in which the efficacy of an intraoperative optimization of selenium status in cardiac surgical patients should be studied. Of note, in a most recent meta-analysis, high-dose supplementation of selenium has been shown to reduce mortality in patients with severe sepsis [31].

Various studies repeatedly indicated ROS to represent one of the major factors contributing to myocardial I/R injury during cardiac surgery [32], [33]. Normally, a sophisticated endogenous defence system including the AOX-enzyme GPx protects tissues from oxidative stress. Assessment of GPx activity was performed to determinate the antioxidant capacity (AOX), hereby indirectly reflecting oxidative stress [7]. The activity of GPx is known to be critically dependent upon circulating selenium levels [9] which is confirmed by our findings of a strong correlation between GPX and selenium in both groups. Interestingly, it has recently been reported that cardiac surgical patients who received a perioperative selenium supplementation showed a reduced extent of myocardial damage [34]. Also in our patients, we could observe an inverse correlation between the postoperatively measured selenium and CK-MB levels. It is tempting to speculate that this inverse correlation may indicate antioxidant and cardioprotective properties of selenium.

ADMA has previously been shown to be crucially involved in the regulation of vascular tone via endothelial nitric oxide synthase (e-NOS) and inducible nitric oxide synthase (i-NOS) [4]. The increased consumption of glutathione during oxidative stress results in increased consumption of homocysteine (Hcy) [35], a potent inhibitor of the ADMA metabolising enzyme dimethylarginine-dimethylaminohydrolase (DDAH) [36]. Thus, a postoperative decrease of Hcy as a consequence of oxidative stress leads to an increased metabolism of ADMA by DDAH. Therefore, ADMA levels decrease with increasing oxidative stress. As we found only an insignificant postoperative reduction in ADMA levels in the on-pump group, we conclude that there was a comparable oxidative stress in both study groups.

The underlying mechanisms of the observed perioperative selenium decrease still have to be elucidated. It has been speculated that selenium levels decrease due to intraoperative blood losses, dilution by resuscitation fluids, extravasation due to systemic inflammation, and depletion owing to the scavenging of reactive oxygen species during/after CPB [12], [37], [38]. We observed a comparable inflammatory response in both groups, as reflected by a comparable perioperative time course of IL-6. This is according to previous studies which demonstrated the surgical trauma itself to represent the main reason for the pro-inflammatory response after both kinds of cardiac surgery [16], [17], [39]. Previous findings in on-pump cardiac surgical patients [4], [40], [41] [42] indicate a higher level of oxidative stress when compared to patients undergoing OPCAB-surgery. In fact, the inevitable use of cardioplegic arrest during conventional on-pump surgery exposes patients to a significant longer duration of myocardial ischaemia and hence more pronounced reperfusion injury when compared to OPCAB-patients where myocardial I/R is minimized by the use of intracoronary shunts during performance of the distal anastomoses. Furthermore, activation of immune cells (e.g. neutrophils and monocytes) after contact with the artificial surfaces of the extracorporeal circuit [5] and the use of mild hypothermia [43] might also contribute to the increased oxidative stress after CPB. Our findings of a comparable level of oxidative stress in both groups do however not support these considerations and do not allow to distinguish the effects of inflammation from those of oxidative stress.

Interestingly, haemodilution and blood loss represent further possible causes for a decrease of circulating selenium levels. Of note, the intraoperative fluid balance differed significantly between our two study groups, most likely due to priming of the extracorporeal circuit with 1500 ml crystalloid fluid. However, postoperative haemoglobin concentration and the transfusion of packed red blood cells (PBRC) were comparable in both groups, suggesting that blood loss and haemodilution contributed only marginally to the observed selenium decreases.

We acknowledge that the present trial suffers from several limitations, including a small sample size, which only allows an adequately analysis of the primary outcome parameter (differences in selenium decrease during two different techniques of coronary artery bypass grafting) with sufficient statistical power. Analyses of the various secondary outcome parameters have to be considered to be purely explorative and hypothesis-generating. A lack of statistical power might also explain why we could not observe differences in the incidence of postoperative organ dysfunction in the OPCAB-group despite a better preservation of the intraoperative selenium status. Furthermore we were unable to clarify whether the observed intraoperative selenium decrease in both groups was truly causative or only “indicative” for increased oxidative stress and the development of organ dysfunction. This question can only be resolved by a large-scale clinical trial in which the efficacy of an intraoperative selenium supplementation strategy has to be tested.

## Conclusion

Cardiac surgery (irrespective of the use of CPB) is associated with an intraoperative decrease of circulating selenium levels. It still remains to be elucidated which mechanisms precisely underlie the intraoperative decrease of selenium levels as both the extent of oxidative stress and inflammatory response were comparable in both groups. Of note, postoperative circulating selenium levels showed a significant predictive accuracy for the occurrence of postoperative organ dysfunction also in OPCAB-patients.

## Supporting Information

Checklist S1 **CONSORT checklist.**
(DOC)

Protocol S1 **Trial protocol.**
(DOC)
