# Supplementary material for: The Importance of Intraoperative Selenium Blood Levels on Organ Dysfunction in Patients Undergoing Off-Pump Cardiac Surgery: A Randomised Controlled Trial
Source: PLoS One. 2014 Aug 13;9(8):e104222. doi: 10.1371/journal.pone.0104222 (PMC4132095; doi:10.1371/journal.pone.0104222)
Supplement: Protocol S1 — Trial protocol. (DOC) [file pone.0104222.s002.doc]

**Influence of cardiopulmonary bypass pump on selenium status of cardiac surgical patients, undergoing coronary artery bypass surgery.**

*Inclusion criteria:*

100 patients, undergoing open heart surgery should be included in the trial. Here, 50 patients will be operated conventional, with the use of CPB and cardioplegia. Further 50 patients will be operated on the beating heart, without the use of CPB (OPCAB= „off pump coronary artery bypass“). The surgeon (Dr. A. K. Menon) will perform the surgery in all patients.

Included will be all patients suffering from a coronary 3-vessels heart disease. They will undergo surgery in general anaesthesia with median sternotomia in the case of elective or urgent indication for treatment. Patients should be free of further serious cardiac diagnoses (valve-disease, aneurysms etc.). Excluded are patients with ischemic cardiomyopathy. The aimed revascularization should be completely performed and conducted by one surgeon. 50 patients are undergoing conventional surgery, with the use of cardiopulmonary bypass (CPB), with moderate hypothermia and cardioplegic cardiac arrest. The other 50 patients will be treated without the use of CPB (OPCAB).

Only adults, after written informed consent for the study participation will be included.

*Exclusion criteria:*

Excluded will be patients with emergency surgery (NSTEMI or STEMI), patients with ischemic cardiomyopathy or additional cardiac diagnoses (valve-diseases or aneurysms), patients incapable of giving consent, pregnant patients and women with childbearing potential, in which pregnancy cannot be excluded.

Patients will be enrolled in the clinical trial, after patient information, at least, the evening before the operation.

*Methodology of study:*

After induction of anaesthesia, within one hour after admission the patient to the intensive care unit and every further morning on the intensive care unit or intermediate care unit (IMC), 10 ml blood will be drawn from the central venous line, placed independently of the study for the operation. A separately puncture for this purpose it is not necessary.

Selenium values will be measured from this blood in the whole blood sample by electrothermal atomic absorption spectroscopy.

Beside this, the following parameters will be measured daily in the routinely diagnostic and later correlated with the selenium concentration at the respective time points. The measurement of glutathione peroxidase (GPX) und thioredoxin (TRX) reductase will be conducted in the laboratory of the company Biosyn. The „macrophage migration inhibitory factor (MIF)“ will be determinated from the remaining blood.

*Determinated parameters:*

- Procalcitonin
- CRP
- IL-6, IL-10
- Leukozyten
- Glutathione peroxidase (GPX)
- Asymmetric dimethylarginin (ADMA)
- Thioredoxin reductase (TRX)
- Platelets
- Bilirubin
- Creatinine
- Lactat
- Albumine
- AT-III
- CK, CKMB and troponin
- Duration of mechanical ventilation
- Duration of hospitalisation
- SOFA-Score
- SAPS-Score

All blood samples will be kept until study end and their analysis (max. 24 months) and will be disposed thereafter.

Values of selenium, markers of oxidative stress (GPX, ADMA) and inflammation (IL-6, IL-10) from the 1.POD on will be used for possible long-term analyses of selenium course.

All data will be recorded on a documentation form.

*Study duration:*

The study will be terminated, as 100 patients are included or after a maximum of 24 months.

*Risks:*

Patients are not exposed to additional risk by blood sampling before surgery, after admission to intensive care unit, and every further day on the intensive care unit. All blood samples will be drawn from catheter, placed independently of study participation, for cardiovascular monitoring. Therefore blood sampling for this study is not associated with pain.

Summarized, according to current scientific evidence there is not expected an impairment or additional stress for the participating patients.

*Benefits:*

There is no benefit for the individual participant of the study expected.

Significant low selenium values (lower than the reference value of selenium in whole blood of 100-140µg/L) will be detected.

*Statistic:*

All data will be recorded pseudonymized and analyzed anonymized.

Recorded patient data will be anonymized, by numbering in order of study inclusion. Thereafter the mean and standard deviation of the recorded data will be calculated and this data will be further statistically evaluated.

Hereby the correlation of measured selenium blood values and laboratory chemical parameters of inflammation and the clinical patient outcome will be evaluated. Differences between the groups (OPCAB vs. conventional CPB-surgery) concerning the laboratory chemical and clinical course will be statistically analyzed by tests comparing independent groups. Correlations between the different variables will be analyzed by the methods of Pearson and Spearman-rho. The predictive value of selenium blood levels concerning certain outcome parameters will be determined by the receiver-operating characteristic curves (ROC).

*Quality assurance:*

This observational study will be conducted under the Good Clinical Practice guidelines.

*Adresses and responsibilities:*

Scientific direction:

PD Dr. Steffen Rex

Senior consultant

Department of Anaesthesiology

University Hospital Aachen

Pauwelsstr. 30

52074 Aachen

Direct line - Tel.: 0049-241-8035186

Tel.: 0049-241-8088179 (secretariat)

Fax: 0049-241-8082406

E-Mail: srex@ukaachen.de

Involved physicians:

Dr. med. A. K. Menon, Senior consultant, Department of Thoracic, Cardiac and Vascular Surgery

Dr. med. C. Stoppe (direct line: 36575), resident physician, Department of Anaesthesiology

Laboratory:

biosyn Arzneimittel GmbH

Schorndorfer Straße 32

D-70734 Fellbach

Telefon: +49 (0)711-57532-00

Telefax: +49 (0)711-57532-99

info@biosyn.de
